# Review of paraneoplastic syndromes associated with oropharyngeal squamous cell carcinoma

**DOI:** 10.4103/0973-029X.72499

**Published:** 2010

**Authors:** Deepu George Mathew, T Rooban, V Janani, E Joshua, UK Rao, K Ranganathan

**Affiliations:** *Department of Oral and Maxillofacial Pathology, Ragas Dental College, Chennai, India*

**Keywords:** Hidden malignancies, oropharyngeal carcinomas, paraneoplastic syndromes

## Abstract

Malignancies are usually preceded by the presence of various paraneoplastic syndromes (PNS), which could be the indirect and/or remote effects of the metabolites produced by neoplastic cells. PNS manifested by oropharyngeal squamous cell carcinomas, which is the most common head and neck malignancy, are highlighted in this review. Knowledge of the clinical spectrum of these syndromes will equip the oral physician for early diagnosis and management of these hidden malignancies, especially of the pharyngeal region.

## INTRODUCTION

The onset of malignancy may be forewarned by a spectrum of clinical manifestations. Several of these findings are manifested in oropharyngeal squamous cell carcinoma (SCC). As these markers may present before an established cancer, dentist should be equipped with the knowledge to recognize the features in order to facilitate appropriate early intervention and management of the neoplasia which helps the patients lead a better quality life.[1]

Paraneoplastic syndromes (PNS) represent a clinical spectrum of manifestations of the indirect and remote effects produced by tumor metabolites or other products and exclude metastasis or any other normal events associated with tumor progression.[2] It is reported that 7.4% of all cancers have PNS associated with them. Although rare, it is important to be aware of these PNS as their clinical presentation could be (i) often the first or prominent clinical manifestation, (ii) can raise suspicion of a deep-seated tumor.[3]

PNS have been reported in patients belonging to all age groups. The symptoms include manifestations occurring as endocrine, dermatological, hematological, neurological, rheumatological and ocular abnormalities. PNS can precede, follow or be concurrent with a malignancy. It important to differentiate these syndromes from false-PNS; which are the symptoms directly related to the invasion of normal tissue by the tumor or by distant metastases.[3]

This article highlights the importance of recognizing PNS caused by oropharyngeal SCC.

## PATHOGENESIS OF VARIOUS PNS

**Figure 1:**
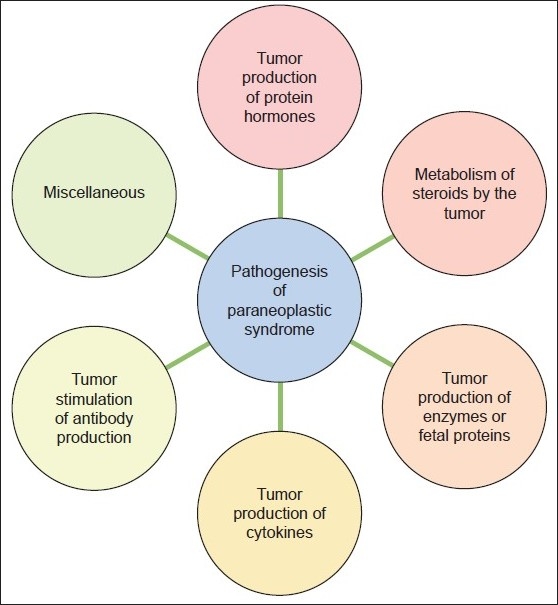
Diagram depicting the various pathways by which paraneoplastic syndromes could occur

The exact nature of the paraneoplastic phenomena associated with underlying malignancy is not fully understood. However, it was suggested that neoplastic cells utilizes more than one way to produce components of PNS. Tumor cells can produce hormones, enzymes or fetal proteins, cytokines, stimulate antibody production and metabolize steroids[4] [Figure 1]. Any of these tumor products can produce manifestations of PNS.

The symptoms of PNS are dependent on their etiopathogenesis, target organ involved, the quantity and quality of secretion of metabolites and other factors. However, certain manifestations are characteristic of a particular group of tumors.Table 1 lists few important symptoms and etiopathogenesis associated with PNS.[5]

**Table 1:** Symptoms and pathogenesis of various PNS in various organs*

Symptom	System	Etiopathogenesis
Fever	Non-specific	Release of endogenous pyrogens; necrotic inflammatory phenomena of tumor; disorders of steroidogenesis
Dysguesia	Non-specific	Alterations in body levels of copper and zinc; morphofunctional variation of papillae
Cachexia	Non-specific	Bioactive molecules produced by the tumor which are able to affect metabolism (increase in the serum levels of fatty acids; decrease of urea, alanine and carbon dioxide; alterations of glucose metabolism)
Hypertrophic osteoarthropathy	Rheumatological	Estrogen or GH production; vagal hyperactivity
Scleroderma and systemic lupus erythematosus	Rheumatological	Release of anti-nuclear antibodies
Nephrotic syndrome	Renal	Secondary kidney amyloidosis; sedimentation of immunocomplexes in nephrons
Neoplastic hypoalbuminemia	Renal	Reduced albumin synthesis
Watery diarrhea	Gastrointestinal	Tumor production of molecules that affect the motility and secretory activity
Malabsorption	Gastrointestinal	Release of prostaglandins by tumor
Protein-losing enteropathy	Gastrointestinal	Tumor mass inflammation and exudation
Erythrocytosis	Hematological	Increase of EPO that results from hypoxia induced by ectopic production of EPO or EPO-like substances or by altered catabolism of EPO itself
Anemia	Hematological	Chronic hemorrhages from ulcerated tumors; altered intestinal absorption of vitamins B-6 and B-12; increased destruction/insufficient production of RBCs; anti-EPO factor; reduction in mean RBC life; poor iron availability
Microangiopathic hemolytic anemia	Hematological	DIC; formation of fibrin filaments in capillary vessels; consequent mechanical hemolysis
Auto immune hemolytic anemia	Hematological	Anti-RBC antibodies
Thrombocytopenia	Hematological	Release of auto-antibodies
Leukemoid reactions	Hematological	Mechanical stimuli on bone marrow, resulting from bone metastases; humoral stimuli resulting from neosynthesized blastic factors or factors released from the foci of tumor necrosis
Leukopenia	Hematological	Metastatic compression on bone marrow
Gammopathies	Hematological	Antigenic stimulus of the tumor on some immune clones
Itching	Dermatological	Hyperesinophilia (typical of Hodgkin’s lymphoma)
Herpes zoster	Dermatological	Reactivation of VZV due to immune system depression
Flushing	Dermatological	Release of prostaglandins and vasoactive substances
Dermic melanosis	Dermatological	Release of melanin precursors into bloodstream
Hypertrichosis	Dermatological	Adrenal dysfunction
Ichthyosis	Dematological	Desquamation of limb surfaces
Hyponatremia	Endocrine	Tumor-producing hormones that affect water and electrolytic balance
Hypocalcemia	Endocrine	Tumor-producing hormones that affect water and electrolytic balance
Hypoglycemia	Endocrine	Production of insulin like growth factor -1 and 2
PNS neuromuscular disorders	Neuromuscular	Latent virus infections becoming virulent; autoantibody formation; production of substances that alter nervous functions
Paraneoplastic encephalitis	Neuromuscular	Lymphocytic infiltration of the medial sections of the temporal lobes, with a loss of neurons
Subacute necrotic myelitis	Neuromuscular	Necrosis of the spinal cord
Eaton-Lambert myasthenic syndrome	Neuromuscular	Production of tumor proteins that may provoke production of anti-calcium-channel antibodies

GH - growth hormone; EPO - erythropoietin; DIC - Diffuse intravascular coagulation; VZV - varicella-zoster virus

* - Adapted from reference 5

### PNS manifestations of oropharyngeal SCCs

#### Endocrine Manifestations

Endocrine symptoms related to PNS usually resemble the more common endocrine disorders.

M Thomson and Adlam DM in their review and case report on ectopic secretion of ADH from the tumor cells associated with oral squamous cell carcinoma (OSCC) have described that the clinical features can range from mild symptoms like fatigue, anorexia and lethargy to severe manifestations like convulsions and coma. This endocrinological manifestation due to the ectopic ADH secretion constitute the syndrome of inappropriate antidiuretic hormone or argentine vasopressin secretion (SIADH). Most patients with SIADH are asymptomatic. SIADH can precede the presentation of cancer by few weeks or months and will recede as the tumor is removed. The persistence of the syndrome despite the treatment for malignancy can be due to continued presence of the tumor.[6] Zerbe *et al*. in their review on inappropriate antidiuresis have mentioned about a small group of patients seen with the same inappropriate antidiuretic state but with no demonstrable elevation of ADH. This was considered to be due to increased sensitivity of the vasopressin receptors or due to the secretion of other vasopressive agents immuonologically distinct from ADH.[7]

Humoral hypercalcemia of malignancy (HHM) is one of the common PNS manifestations of head and neck SCCs [Table 2]. Iwase M *et al*.[8] in their study on 242 patients of OSCC found that 5% of them showed hypercalcemic complications. The patients with malignancy-associated hypercalcemia had a biochemical profile similar to hyperparathyroidism,[9] but in many cases the parathyroid hormone (PTH) levels were not elevated. Hypercalcemic state is found to be due to parathyroid hormone-related protein (PTH-rP). Rankin W *et al*. in their review on PTH-rP and hypercalcemia have mentioned that the receptor binding and activation domains of PTH and PTH-rP are present with in the first 34 amino acids and both the hormones have identical actions of inducing hypercalcemia by promoting bone resorption and decreasing calcium excretion.[10] Hypercalcemia can induce clinical manifestations like central nervous system depression, muscular weakness, cardiac abnormalities, gastrointestinal disturbances and renal failure. It is one of the important causes of morbidity and mortality in patients with cancer. The mean survival time after the diagnosis of HHM in a patient with cancer is usually 54.9 days, hence development of hypercalcemia in patients with cancer signals grave prognosis.[9]

Imura *et al*. in his review on ectopic ACTH-producing tumors have reported a case of 66-year-old man with laryngeal SCC. One year after the excision, metastasis in the liver was found associated with chemical hyperadrenocorticism. Bioassayable ACTH and MSH were detected in the metastatic tissue.[11]

Paraneoplastic gynecomastia was reported by Scholl *et al*. in a 47-year-old man with poorly differentiated OSCC. Patient underwent surgery and radiotherapy but later complained of bilateral breast enlargement and tenderness. Serum β-HCG level was found to be elevated. Later the patient developed axillary node positivity for metastasis. The tumor tissues in the primary and metastatic site were positive for β-HCG. It is reported that such β-HCG-producing tumors usually have poor prognosis.[12]

#### Neuromuscular manifestations

Ferlito A and Rinaldo A in their review have mentioned that cases of laryngeal SCC has been reported to manifest subacute cerebellar degeneration due to antineural antibodies produced by the tumor cells causing neuronal death.[12] Owing to the degeneration; in-coordination first appears in legs causing abnormal stance and unsteady gait of wavering type, disturbance in the speech articulation, vertigo, intentional tremor, ataxia, ocular disturbances, nystagmus, ocular dysmetria and opsoclonus. Thus, whenever one encounters any non-familial ataxia arising in patients of fourth to fifth decade, an underlying malignancy has to be considered and ruled out. These manifestations are often seen within weeks of the occurrence of cancer.[13]

Ferroir JP *et al*. and Shipley E *et al*. have reported cases of laryngeal SCC associated with a neurologic PNS called Eaton-Lambart myasthenic syndrome. This PNS usually occurs due to auto antibodies released by tumor cells reducing the release of acetylcholine at the neuromuscular junction causing weakness, myalgias, fatigability especially in the lower extremities and proximal muscles. It is a disorder associated with dysautonomic symptoms that include dryness of mouth and eyes, impotence, diminished sweating and orthostatic symptoms. The symptoms are reported to regress on the removal of the tumor.[14]

Baijens LWJ and Manni JJ in their report of four head and neck cancers associated with PNS has described a case of laryngeal SCC with neck metastasis manifesting paraneoplastic encephalomyelitis. The patient was found to have anti-Hu-antibodies which were directed against the nuclei of central nervous system cells causing inflammation of the grey matter in central nervous system. This disorder consists of five entities determined by the anatomic site of the lesion - they are cerebral encephalitis, brain stem encephalitis, cerebellar encephalitis, subacute myelitis and dorsal root ganglionitis. The brain stem encephalitis typically shows vertigo ataxia, nystagmus, nausea and emesis, when spinal cord is involved symptoms resembling poliomyelitis are seen due to neuronal loss in the anterior and posterior horn of spinal cord. Anti-Hu-positive paraneoplastic encephalomyelitis in association with supraglottic SCC has been reported;[15] another case of paraneoplastic limbic encephalitis was reported in association with piriform sinus SCC.[16]

#### Ocular manifestations

Laryngeal SCC associated with photophobia was reported by CE Prac *et al*.[17] in which auto antibodies directed against retinal antigens were demonstrated. Carcinoma-associated retinopathy occurs due to the expression of retinal antigens like recovering by the malignant cells and an autoimmune reaction mounted against these antigens. The autoantigens penetrate retinal cells and induce apoptosis. It is clinically characterized by the progressive loss of photoreceptors leading to painless vision loss with night blindness, light-induced glare, photosensitivity and peripheral and ring like scotomas.[18]

#### Rheumatological manifestations

Polyarthritis has been reported to present as a PNS. The typical features of that can hint the presence of a hidden malignancy are asymmetric joint disease, explosive onset, predominant involvement of the legs with sparing of the wrists and small joints of the hands with absence of rheumatoid factor, rheumatoid nodules and a family history of rheumatoid arthritis. A sequential relationship should be present between the onset of the articular symptoms and detection of the tumor. The presence of hypertrophic osteoarthropathy or metastatic involvement of synovium or periarticular bone should be excluded.[13] These symptoms are often reported to precede the diagnosis of the tumor by months. There are few reported cases of laryngeal and pharyngeal SCC-associated polyarthritis.[19] Eggelmeijer and Macfarlane reported a case manifesting polyarthritis along with the occurrence of primary laryngeal SCC carcinoma which regressed on tumor removal but manifested again with recurrence.[20]

A case of polymyalgia rheumatica manifested as stiffness and pain in shoulder and hip muscles associated with a hypopharyngeal SCC has been reported in the German literature.[19] Ferlito *et al*. in his review on PNS have described about pseudo-stills disease which is an inflammatory arthritis characterized by swelling, tenderness and pain in one or more joint with lymphnode or splenic enlargement. There are few reported cases of laryngeal carcinoma associated with this manifestation.[2]

Hypertrophy osteoarthropathy is a disorder characterized by clubbing of digits, periosteal new bone formation and arthritis. When associated with malignancy they tend to have a rapid onset and progression. They often precede the clinical manifestations of the hidden tumor by months. Deep seated pain in the distal extremities is usual due to periostitis. In patients with malignancies, severe skeletal pain is found before the appearance of clubbing. Few cases of hypopharyngeal SCC are reported to occur with this condition. Makenzie and Scherbel have reported two such cases of laryngeal SCC in their review.[21]

#### Dermal manifestations

Itching is the most frequent cutaneous manifestation in patients with cancer. Herpes zoster and alopecia presenting as part of a PNS are similar to their equivalent benign forms. Immune system depression, which may be observed in most patients with cancer, is often responsible for the reactivation of latent varicella-zoster virus (VZV) in the sensory ganglia.[4]

Acanthosis nigricans is a common paraneoplastic manifestation of skin frequently associated with abdominal adenocarcinoma. It is characterized by light or dark areas of hyperpigmentation and warty lesions usually affecting the flexural surfaces. Oral lesions appear as diffuse fine papillary areas most often involving tongue and lips.[22] Oppolzer G *et al*. and Miller TR *et al*. have reported cases of laryngeal SCC associated with this dermatological PNS.[2]

Acrokeratosis Paraneoplastica (Bazex syndrome), is a PNS with dermatological manifestations exclusively associated with malignancies of upper aerodigestive tract including OSCC and metastatic cervical lymph nodes. Miquel JF *et al*. in his report of a case of laryngeal SCC with Bazex syndrome has described the clinical spectrum of this PNS. In the initial stages when tumor is not apparent, the dermatosis is characterized by the appearance of violacious erythema and slight psoriasiform scaling that appears on the hands and feet (acral sites). Nail dystrophic changes and involvement of aural helixes and nose occur in early stages. Eventually in advanced stages, psoriasiform plaques develop extending centripetally affecting other areas like legs, knees, thighs, arms and trunks. In these advanced stages usually the clinical symptoms of the tumor becomes apparent.[23] The pathogenesis is described in [Table 2]. Most of the associated cases are SCC and these manifestations often appear before the clinical features of the tumor become apparent. The interval between the onset of skin manifestations and diagnosis of malignancy is usually 8-11 months. There is often a rapid improvement of the dermatosis upon the removal of the underlying tumor.[24]

**Table 2:** PNS associated with oropharyngeal carcinomas

Condition	Prevalence	Suggested etiology	References
Schwartz-barter syndrome	2-3%	Ectopic secretion of arginine vasopressin	Schwartz WB *et al*., 1957
		Lysis of cancer cells releasing vasopressin or analog proteins	Hayes *et al*., 1986
		After neck dissection, modified venous return after ligation of internal jugular vein resulted an increase in intracranial pressure leading to SIADH	McQuarrie *et al*., 1977 Wenig and Heller, 1987,
		Direct invasion of the vagus nerve leading to baroreceptor denervation which may cause hyponatremia	Zerpe R *et al*., 1980
Humoral hypercalcemia	2.6-7.2%	Serum parathyroid hormone-related peptide (PTH-rP) is secreted by several tumors of epithelial origin. False – serum phosphate is normal/increased, elevated calcium attributed to dissolution of bone by metastasis; True – elevated levels of PTH-rP cross-react with parathyroid hormone receptors, promoting bone resorption and increasing distal renal tubular reabsorption	Pande SB *et al*., 2007
Humoral hypercalcemia with leukocytosis	2.2%	Granulocytes and osteoclasts share a common hematopoietic precursor, hence tumor factors that stimulate osteoclast formation could also stimulate granulocyte formation	Yoneda T *et al*., 1991
Bazex syndrome	Not available	Autoimmune reaction possibly initiated and triggered by a common antigen between tumor and epidermal cells.	Sarkar *et al*., 1998
		Stimulating effect of TNF–α produced by tumor cells	
PNS Gynecomastia	Rare	Increased β-HCG production by tumor cells	Scholl *et al*. 1997
Malignancyassociated Sweet’s syndrome	Rare	Tumor-associated production of granulocyte colony-stimulating factor can be responsible for the marked leukocytosis and consequent neutrophillic dermatosis.	Reina C *et al*., 1998
Digital Necrosis	Rare	Either related to arterial vasospasm mediated by tumor immune complexes or due to the action of the antibodies against the tumor antigens inducing digital vasculitis.	Wright JR *et al*., 2002
Yellow nail syndrome	Rare	The anatomical or functional abnormalities of the lymphatic draining system are considered to be responsible for this disorder	Guin JD *et al*., 1979
Paraneoplastic pemphigus	Rare	Auto-antibodies against the desmosomal and hemidesmosomal components of oral epithelium	Wong KC *et al*., 2000
Trosseau syndrome	Rare	Hypercoagulable material released from the tumor	Ferlito A *et al*., 2007

PNS – Paraneoplastic syndromes; TNF– α - Tumor Necrosis Factor; β-HCG - Human Chorionic Gonadotrophin

Sweets syndrome (acute febrile neutrophillic dermatosis) as reported by Dawe SA *et al*. and Renia CC *et al*. can occur as a PNS of oropharyngeal SCCs. Cohen PR has described the clinical and histopathologic manifestations of Sweets syndrome in his review. Fever is the most frequent symptom but cutaneous manifestations will precede the fever by days to weeks. Skin lesions have a symmetrical distribution, painful and appear as purple/red plaques/nodules or as plaques. These lesions have a transparent vesicle like appearance due to the edema in the upper dermis, some of them can get ulcerated and resemble pyoderma gangrenosum. It is also associated with skin pathergy or skin hypersensitivity which is characterized by the appearance of dermatosis at the site of cutaneous trauma. These include areas of biopsies, IV catheter placement, areas that have received radiation therapy and areas of sunburn. Histopathologically the lesions are characterized by dense neutrophil infiltrate in the upper dermis with edema. The overlying epidermis is normal and changes of primary leukocytoclastic vasculitis are usually absent.[25,26]

Wright JR and Gudelis S from Regional Cancer Centre, Ontario has reported a case of SCC of tonsil associated with digital necrosis as a PNS which was characterized by acral ischemia and multiple necrosis of the finger tips associated with severe pain.[27] Yellow nail syndrome is a rare disorder characterized by the triad of yellow and thickened nails, lymphedema and respiratory manifestation commonly pleural effusion and other complications like bronchiectasis and chronic sinusitis. Lymphedema in yellow nail syndrome is characteristically non-pitting and involves the lower extremities in symmetric fashion. The associated edema has also been described in the upper extremities especially in the face.[28] Ferlito A and Rinaldo A in their review of PNS associated with head and neck cancers has described a patient with laryngeal SCC having yellow nail syndrome as PNS, which regressed after the excision of the tumor.[29]

Paraneoplastic pemphigus is usually associated with autoantibodies from tumor cells against the desmosomal and hemidesmosomal components of epithelium. Occurrence of this disease has been reported in association with tongue SCC by Wong KC and HO KK.[30] Paraneoplastic pruritus is a generalized dermatological manifestation seen along with hematological neoplasms like Hodgkin’s lymphoma and other solid tumors and rarely reported in oropharyngeal SCCs. A case of pruritis associated with SCC of the larynx was reported by Rantuccio F. Necrolytic migratory erythema is a skin disorder characterized by skin rashes on the legs, perineum and groin that begin as an erythema and progress to form superficial blisters and spreads with central clearing. Even after resolution, areas of acute eruptions remain indurated and hyperpigmented. Glossitis, stomatitis, dystrophic nail changes and hair thinning are also reported along with this syndrome. This paraneoplastic manifestation is usually a part of a rare syndrome comprising of diabetes mellitus with pancreatic islet cell tumor referred as glucagonama syndrome. If not associated with pancreatic islet cell tumor it’s called psuedoglucagonoma syndrome. A case of hypopharyngeal SCC with persistent disseminated necrolytic migratory erythema for 8 months with no underlying glucagonoma is reported by Mohrenschlager *et al*.[29]

#### Hematological manifestations

Spontaneous recurrent or migratory episodes of venous thrombosis, arterial emboli due to non-bacterial thrombotic endocarditis are seen as a PNS in a patient with underlying malignancy. This is due to hypercoagulable materials released from the tumor cells. These thrombotic episodes respond to heparin but not to warfarin. This PNS is known as Trousseaus’s syndrome. They are usually associated with gastric carcinomas but rarely observed in laryngeal carcinomas.[29]

Okada M *et al*. reported a case of paraneoplastic polyvasculitis in a 66-year-old patient with underlying hypopharyngeal SCC along with cyanosis, gangrene in the bilateral fingers and toes and pleural effusion. Arteriography revealed several occluded arteries.[31] Leukocytosis, has been documented in a 54-year-old man presenting with SCC of tongue. These leukemoid reactions can be due to the humoral stimuli produced by the blastic factors synthesized from the foci of tumor necrosis.[19]

Symptoms related to erythrocytosis or anemia, thrombocytosis, disseminated intravascular coagulation (DIC), and leukemoid reactions, characterized by the presence of immature WBCs in the bloodstream, are usually accompanied by hypereosinophilia and itching. Thrombocytosis (>500,000 platelets/dl), erythrocytosis and anemia are certain non-specific diseases produced by tumors. Anemia is frequently the presenting symptom of several neoplasms and results from chronic hemorrhages from ulcerated tumors, altered intestinal absorption of vitamins B-6 and B-12, and increased destruction or insufficient production of RBCs. Three types of PNS anemias are described in the literature: (i) Chronic anemia resulting from an anti-erythropoietin factor (ii) microangiopathic hemolytic anemia resulting from DIC, with formation of fibrin filaments in capillary vessels and consequent mechanical hemolysis (iii) Autoimmune hemolytic anemia resulting from anti-RBC antibodies that can be produced by antibodies directed against novel antigens produced by tumors. DIC is typical of epithelial tumors, leukemias and lymphomas (in particular, acute promyelocytic leukemia).Their occurrence in OSCC, however, is rare and rather non-specific.[5]

## DISCUSSION

**Figure 2:**
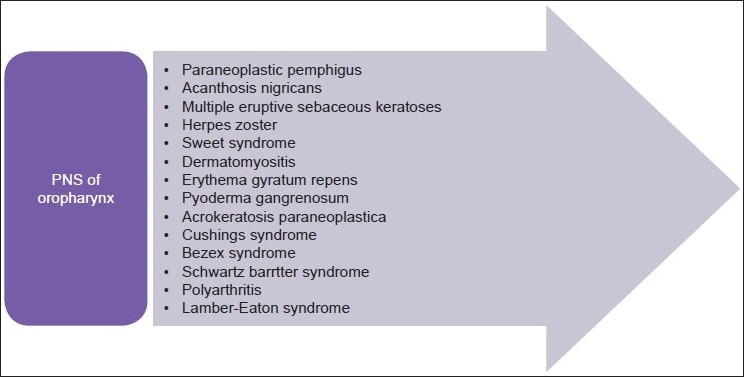
PNS associated with oropharyngeal SCCs

PNS are conventionally described as systemic and non-metastatic manifestations associated with a variety of malignancies. They often produce signs and symptoms remote from the site of a malignant tumor, resulting from damage to organs or tissues by mechanisms that are not fully understood but fit into categories as described in Figure 2.

PNS caused by oropharyngeal SCC could manifest in a variety of ways described above. They can precede, follow or be concurrent with the diagnosis of SCC, although the diagnosis of PNS is challenging owing to overlap of signs and symptoms. Interestingly, to the best of our knowledge and recent reports, only two (endocrine and dermatologic) of the six main groups of PNS have been reported frequently in association with oral cancer. Humoral hypercalcemia is the most frequent, but it is, in most of the cases, a late manifestation of the oral cancer. Thus it has an important negative prognostic value. Dermatological PNS are less common, but may be a premonitory sign, as they frequently precede the oral tumor. Awareness of this association is important as the first indicator of a malignant tumor or its recurrence.

## CONCLUSION

A rapid and correct diagnosis of any PNS may lead to better prognosis. Early recognition of these PNS may lead to clues about the underlying conditions and this may help to avoid diagnostic errors and permit earlier diagnosis and better early intervention. PNS may evolve over weeks to months (more rarely, 1-3 year) and may stabilize, regardless of whether the patient’s symptoms improve or get worse. This article aims to highlight the importance of recognizing PNS associated with oropharyngeal SCCs. Dentists may play an extremely valuable role in the early identification of these conditions through familiarity with these manifestations. Ultimately, timely detection of the cancer-associated syndromes can have an enormous impact on the quality of life and prognosis of this patient population.
